# Genotype-phenotype pattern analysis of pathogenic *PAX9* variants in Chinese Han families with non-syndromic oligodontia

**DOI:** 10.3389/fgene.2023.1142776

**Published:** 2023-03-28

**Authors:** Jiabao Ren, Sifang Gan, Shushen Zheng, Meikang Li, Yilin An, Shuo Yuan, Xiuge Gu, Li Zhang, Yan Hou, Qingqing Du, Guozhong Zhang, Wenjing Shen

**Affiliations:** ^1^ Department of Prosthodontics, Hebei Key Laboratory of Stomatology, Hebei Clinical Research Center for Oral Diseases, School and Hospital of Stomatology, Hebei Medical University, Shijiazhuang, China; ^2^ Xingtai Medical College, Xingtai, China; ^3^ Department of Orthodontics, Hebei Key Laboratory of Stomatology, Hebei Clinical Research Center for Oral Diseases, School and Hospital of Stomatology, Hebei Medical University, Shijiazhuang, China; ^4^ College of Forensic Medicine, Hebei Medical University, Shijiazhuang, China; ^5^ Hebei Key Laboratory of Stomatology, Hebei Clinical Research Center for Oral Diseases, School and Hospital of Stomatology, Hebei Medical University, Shijiazhuang, China

**Keywords:** tooth agenesis, non-syndromic oligodontia, PAX9 gene, whole-exome sequencing (WES), functional analysis, phenotype

## Abstract

**Background:** Non-syndromic oligodontia is characterized by the absence of six or more permanent teeth, excluding third molars, and can have aesthetic, masticatory, and psychological consequences. Previous studies have shown that *PAX9* is associated with autosomal dominant forms of oligodontia but the precise molecular mechanisms are still unknown.

**Methods:** Whole-exome and Sanger sequencing were performed on a cohort of approximately 28 probands with NSO, for mutation analysis. Bioinformatic analysis was performed on the potential variants. Immunofluorescence assay, western blotting, and qPCR were used to explore the preliminary functional impact of the variant PAX9 proteins. We reviewed *PAX9*-related NSO articles in PubMed to analyze the genotype-phenotype correlations.

**Results:** We identified three novel *PAX9* variants in Chinese Han families: c.152G>T (p.Gly51Val), c.239delC (p.Thr82Profs*3), and c.409C>T (q.Gln137Ter). In addition, two previously reported missense variants were identified: c.140G>C (p.Arg47Pro) and c.146C>T (p.Ser49Leu) (reference sequence NM_006194.4). Structural modeling revealed that all missense variants were located in the highly conserved paired domain. The other variants led to premature termination of the protein, causing structural impairment of the PAX9 protein. Immunofluorescence assay showed abnormal subcellular localizations of the missense variants (R47P, S49L, and G51V). In human dental pulp stem cells, western blotting and qPCR showed decreased expression of PAX9 variants (c.140G>C, p.R47P, and c.152G>T, p.G51V) compared with the wild-type group at both the transcription and translation levels. A review of published papers identified 64 PAX9 variants related to NSO and found that the most dominant feature was the high incidence of missing upper second molars, first molars, second premolars, and lower second molars.

**Conclusion:** Three novel *PAX9* variants were identified in Chinese Han families with NSO. These results extend the variant spectrum of *PAX9* and provide a foundation for genetic diagnosis and counseling.

## 1 Introduction

Tooth agenesis (TA) is a common developmental craniofacial anomaly with aesthetic, masticatory, and psychological consequences (Yu et al., 2019). TA may occur as syndromic TA, as part of multiple congenital anomalies, or as non-syndromic TA that only affects dentition (Yu et al., 2019; Zeng et al., 2019). The prevalence of non-syndromic TA ranges from 3% to 10% among different ethnicities and geographic areas (Letra et al., 1993). Based on the number of permanent missing teeth (excluding third molars), TA can be classified as hypodontia (fewer than six missing teeth), oligodontia (six or more missing teeth), and anodontia (complete edentulism) (Fauzi et al., 2018). Oligodontia is a more severe and less common form than hypodontia, with a prevalence of 0.1%–0.5% (Letra et al., 1993; Wong et al., 2018), and compelling evidence shows that the most significant factor for its pathogenesis is genetic (Ye and Attaie, 2016; Choi et al., 2017).

More than 300 genes are involved in different phases of tooth development^1^ (Wong et al., 2018). At least eight genes have been identified as the major causes of non-syndromic oligodontia (NSO): *MSX1* (Zheng et al., 2021), *PAX9* (Sun et al., 2021), *AXIN2* (Wong et al., 2014), *WNT10A* (Arzoo et al., 2014), *LRP6* (Massink et al., 2015), *EDA* (Zhang et al., 2020), *EDAR* (Zhang et al., 2021), and *WNT10B* (Yu et al., 2016). Among these, *PAX9* is a critical transcription factor expressed in the prospective tooth mesenchyme prior to any morphological manifestation and initiates tooth development (Peters et al., 1998a; Wong et al., 2018). In *PAX9*-deficient mice, tooth development is arrested at the bud stage, and the condensation of mesenchymal cells around the bud is less prominent compared with the wild type. This implies a failure of the mesenchyme to induce the epithelial signaling center, the primary enamel knot needed for tooth morphogenesis, and advancement from the bud-to-cap stage (Peters et al., 1998a; Nieminen, 2009; Balic and Thesleff, 2015).

*PAX9* was among the earliest genes to be associated with autosomal dominant forms of oligodontia in humans (Stockton et al., 2000). *PAX9*, located on human chromosome 14q13.3, contains 1,026 bases and encodes a member of the paired box family of transcription factors. The protein has a total of 341 amino acids and is composed of a paired domain (PD) and an octapeptide motif (OM). To date, more than 50 pathogenic variants have been reported in *PAX9* that lead to NSO (Sun et al., 2021). Most *PAX9* variants cluster in and around the paired domain, indicating that this region might be a mutation hotspot; but the precise pathogenic mechanism of *PAX9*-related NSO is still unknown. A review of published papers revealed that haploinsufficiency of PAX9 may be involved (Nieminen et al., 2001; Klein et al., 2005; Mostowska et al., 2006; Liang et al., 2016), while others suggest dominant-negative effects of *PAX9* variants (Sun et al., 2021). In mouse models, dose effects of the Pax9 gene through downregulation of mRNA transcription are shown to influence the severity of oligodontia (Kist et al., 2005).

In this study, we identified three novel *PAX9* variants and two reported variants in the Chinese Han family. *In silico* and preliminary functional analyses were used to predict the pathogenicity of the detected variants.

## 2 Materials and methods

### 2.1 Enrollment of subjects and ethical approval

A cohort of 28 non-consanguineous probands with NSO, who had been referred to the Department of Prosthodontics at Hebei Medical University Hospital of Stomatology, and 100 non-consanguineous controls without TA were recruited. All probands were examined by prosthodontics specialists to determine their physical and intraoral status, and panoramic radiographs were obtained to verify tooth agenesis. All probands asserted that their missing permanent teeth were congenital and were not caused by injury or extraction. This project was approved by the Ethics Committee of the School and Hospital of Stomatology, Hebei Medical University [No: (2016)004] and carried out in accordance with the principles of the Declaration of Helsinki. Written informed consent was obtained from all participants.

### 2.2 Variant detection

Genomic DNA of 28 patients, their available family members, and 100 controls was isolated from peripheral blood using the E.Z.N.A. Blood DNA Midi Kit (Omega Bio-Tek Inc., Norcross, GA, United States), according to the manufacturer’s instructions. Whole-exome sequencing was performed only for probands with NSO. In brief, DNA libraries were prepared using the Fast Library Prep Kit (iGeneTech Bioscience Co., Ltd., Beijing, China), according to the manufacturer’s instructions, and sequenced on the Illumina NovaSeq 6,000 platform (Illumina, San Diego, CA, United States) by the iGeneTech Institute. Sequence reads were mapped to the human reference genome hg19 (GRCh37) using the Burrows-Wheeler Aligner (v.0.7.17) (Li and Durbin, 2009). Small indels and single-nucleotide variants were identified using SAMtools (Danecek et al., 2021) and the Genome Analysis Toolkit (GATK) before annotation using ANNOVAR.

Variants were filtered to include only those in exonic regions or within splice site regions and those with minor allele frequencies (MAF) <1% in dbSNP, the 1000 Genomes Project databases, Exome Aggregation Consortium (ExAC), and Genome Aggregation Database (gnomAD), and to exclude synonymous variants. The variants were verified by PCR amplification of the candidate *PAX9* gene (reference sequence GenBank NM_006194.4). The PCR products were sent to Sangon Biotech Company for purification and Sanger sequencing. Prediction of the effects of the candidate *PAX9* variants was performed using Sorting Intolerant from Tolerant (SIFT) (Sim et al., 2012), PolyPhen 2 (Adzhubei et al., 2010), and Mutation Taster (Schwarz et al., 2014).

### 2.3 Homology analysis and protein structural modeling analysis

COBALT^2^ was used to perform multispecies amino acid sequence alignment of PAX9 and multiple sequence alignment among the PAX families of *Homo sapiens*. Swiss-model (Waterhouse et al., 2018) and PyMOL (Schrödinger) was used to visualize the three-dimensional structure of the PAX9 variants. I-Mutant3.0^3^ as used to predict stability changes in the protein caused by a single point protein mutation.

### 2.4 Plasmid construction

To construct the wild-type plasmid, the full coding sequence of wild-type *PAX9* was subcloned into the expression vector pCMV-C-Flag (Beyotime Biotechnology, Haimen, Jiangsu, China) between HindIII and XbaI restriction sites. Three missense variants of *PAX9* (c.140G>C, c.146C>T, and c.152G>T) were generated using site-directed mutagenesis. A 3′-XbaI primer was designed prior to the premature stop codon and a 5′-HindIII primer at the start codon to amplify truncated fragments by PCR. The fragments were then subcloned into the expression vector in front of the Flag-epitope. All plasmids constructed were verified by Sanger sequencing.

### 2.5 Cell culture, transient transfection, and immunofluorescence

Human dental pulp stem cells (hDPSCs) (Beijing Tason Biotech) were cultured in Dulbecco’s modified Eagle medium (Gibco) with 10% fetal bovine serum (Gibco) at 37°C in a humidified atmosphere. Lipofectamine 3,000 Transfection Reagent (ThermoFisher) was used for transient transfections. Forty-eight hours after transfection, hDPSCs were fixed with 4% paraformaldehyde, permeabilized using 0.25% Triton-X100(Gibco), stained with 4′,6-diamidino-2-phenylindole(Abcam) and mounted. Images were taken using an LSM 510 Meta confocal microscope (Leica Camera AG, Wetzlar, Germany) with a ×20/2.00 objective lens.

### 2.6 Western blotting, q-PCR, and statistical analysis

Western blot analysis was performed using 35 μg of protein from the cell lysates. Total protein was transferred to polyvinylidene difluoride (PVDF) membrane (Bio-Rad). The membrane was incubated with the following primary antibodies: anti-FLAG (Sigma, St Louis, MO, United States) and anti-GAPDH (Beyotime). Protein bands were visualized using ECL reagent (Thermo) and imaged using ImageJ software (Schneider et al., 2012).

Total RNA from hDPSCs was extracted using an RNA extraction kit Trizol(Beyotime), and cDNA was synthesized using a PrimeScript RT Reagent Kit with gDNA Eraser (Takara Bio Inc., Kusatsu, Shiga, Japan). Specific primer sequences were used to amplify targets for GAPDH (5′-TGGAGCCAAAAGGGTCA-3′and 3′-CTT​CTG​GGT​GGC​AGT​GA-5′) and the quantity of the target was normalized to that of GAPDH.

Experiments were performed in triplicate. Data are presented as mean ± SD. The significance of differences was analyzed using a one-way analysis of variance or Student’s *t*-test. Statistical analyses were performed using SPSS 21.0 software (IBM Corporation, Armonk, NY, United States). For all comparisons, two-sided *p* < 0.05 was considered statistically significant.

### 2.7 Literature review

We identified papers reporting *PAX9* variants in PubMed, published until 2022.12.31, for phenotype-genotype analysis. Out of 241 papers, a total of 193 articles without specific variant sites, specific tooth agenesis phenotypes, synonymous variants, or SNPs were discarded. Thus, 48 articles describing 193 patients were included in the analysis.

## 3 Results

### 3.1 Clinical findings and identification of three novel variants of *PAX9*

**FIGURE 1:**
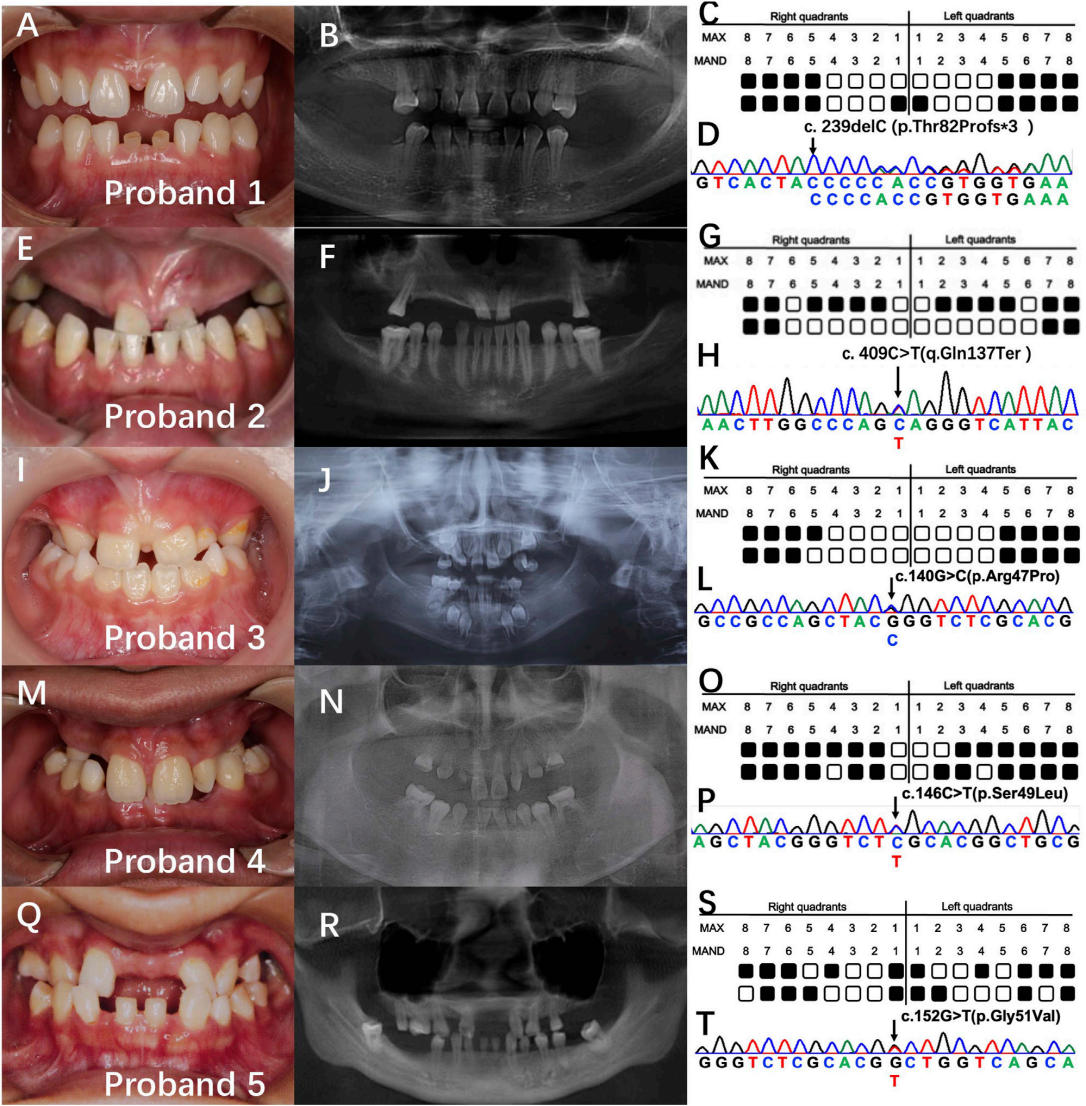
Clinical findings and genetic analysis of five probands. **(A, E, I, M, Q)** intraoral photos. **(B, F, J, N, R)** panoramic radiographs. **(C, G, K, O, S)** schematics of the tooth missing pattern of the proband. Black squares indicate missing tooth positions. **(D, H, L, P, T)** sequencing chromatograms of the proband.

Screening of mutations in 28 non-consanguineous patients with NSO revealed five probands (16.7%) with distinct *PAX9* mutations. The number of missing teeth, excluding the third molars, ranged from 11 to 21; with a mean of 14.6 (Figures 1C, G, K, O, S). Probands 2–5 were sporadic, and proband 1 had a familial background. All showed an autosomal dominant inheritance pattern. Panoramic radiographs (Figures 1B, F, J, N, R) show that agenesis of the molars was most prevalent (37/40), followed by that of the premolars (18/20). Proband 2 showed skeletal Class III malocclusion. Proband 5 showed a conical maxillary second incisor. No additional anomalies, such as facial appearance, hair, skin, nail, lacrimal, sweating, or salivary secretions, were identified in the probands and their families.

Whole-exome sequencing resulted in approximately 18,462 Mb of raw data per individual, with a mean target depth of 145x, and was prioritized based on the above-mentioned parameters. We identified five *PAX9* variants in exon 3 including three novel variants: proband 1: c.239delC (p.Thr82Profs*3), proband 2: c.C409T (q.Gln137Ter), and proband 4: c.152G>T (p.Gly51Val). Two reported variants were identified: proband 3: c.140G>C (p.Arg47Pro) and proband 5: c.146C>T (p.Ser49Leu) (Figure 1D). Furthermore, the three novel variants were not found in healthy controls (*n* = 100) or ExAC and 1,000 Genomes databases, indicating that they were rare. All variants were predicted to be disease-causing by *in silico* algorithms, including SIFT, PolyPhen-2, and Mutation Taster.

### 3.2 Homology analysis and conformational analysis of *PAX9* variants

**FIGURE 2:**
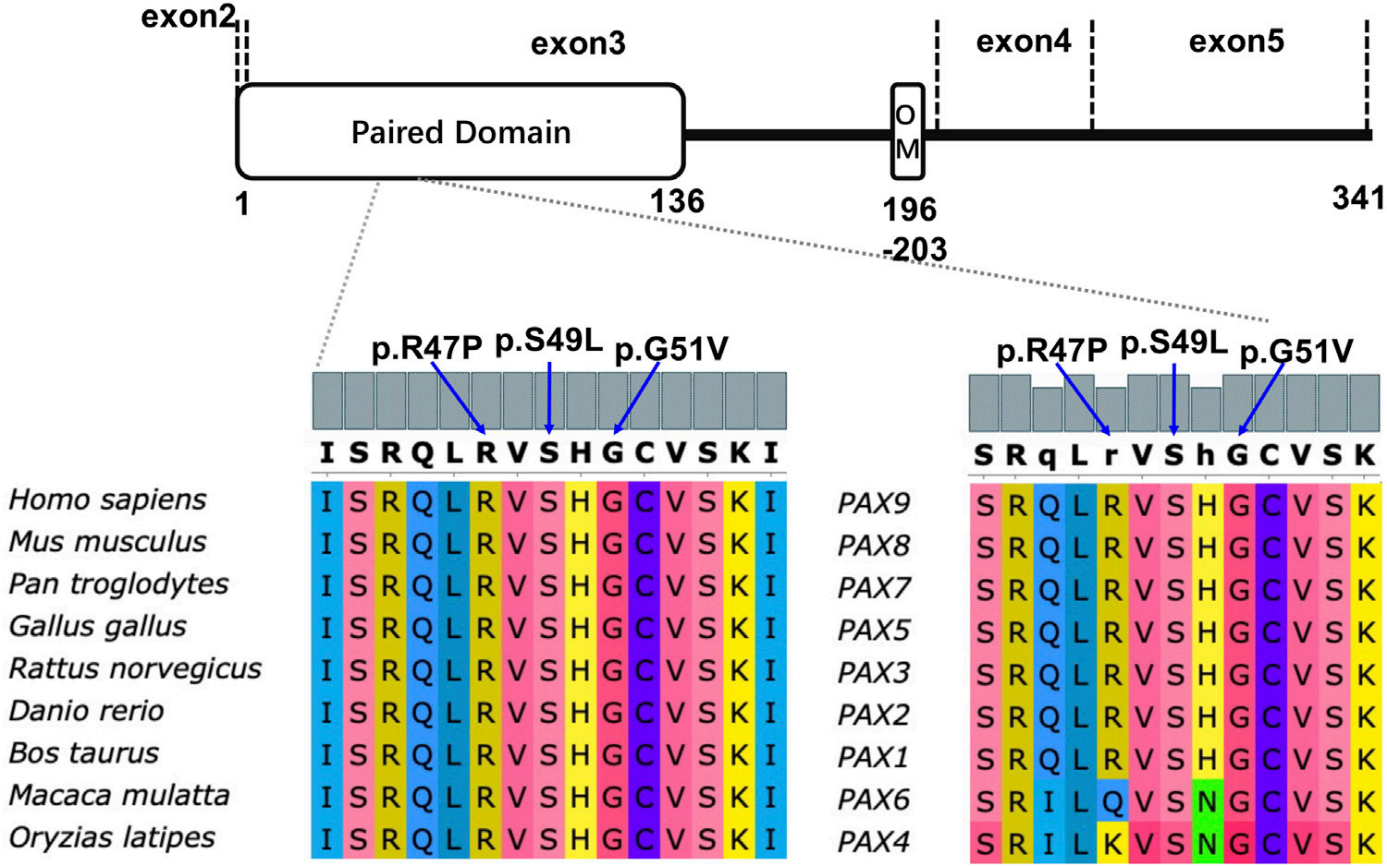
Schematic diagram of PAX9 protein (NP_006185.1) and conservation analysis of affected amino acids in paired domain among nine different vertebrate species and all PAX paralogs of *homo sapiens*.

Multiple sequence alignment showed that the affected residues 47Arg, 49Ser, and 51Gly in *PAX9* variants were located in the PD in exon 3. This is evolutionarily conserved across *Pax9* orthologs and related paralogs (Figure 2).

**FIGURE 3:**
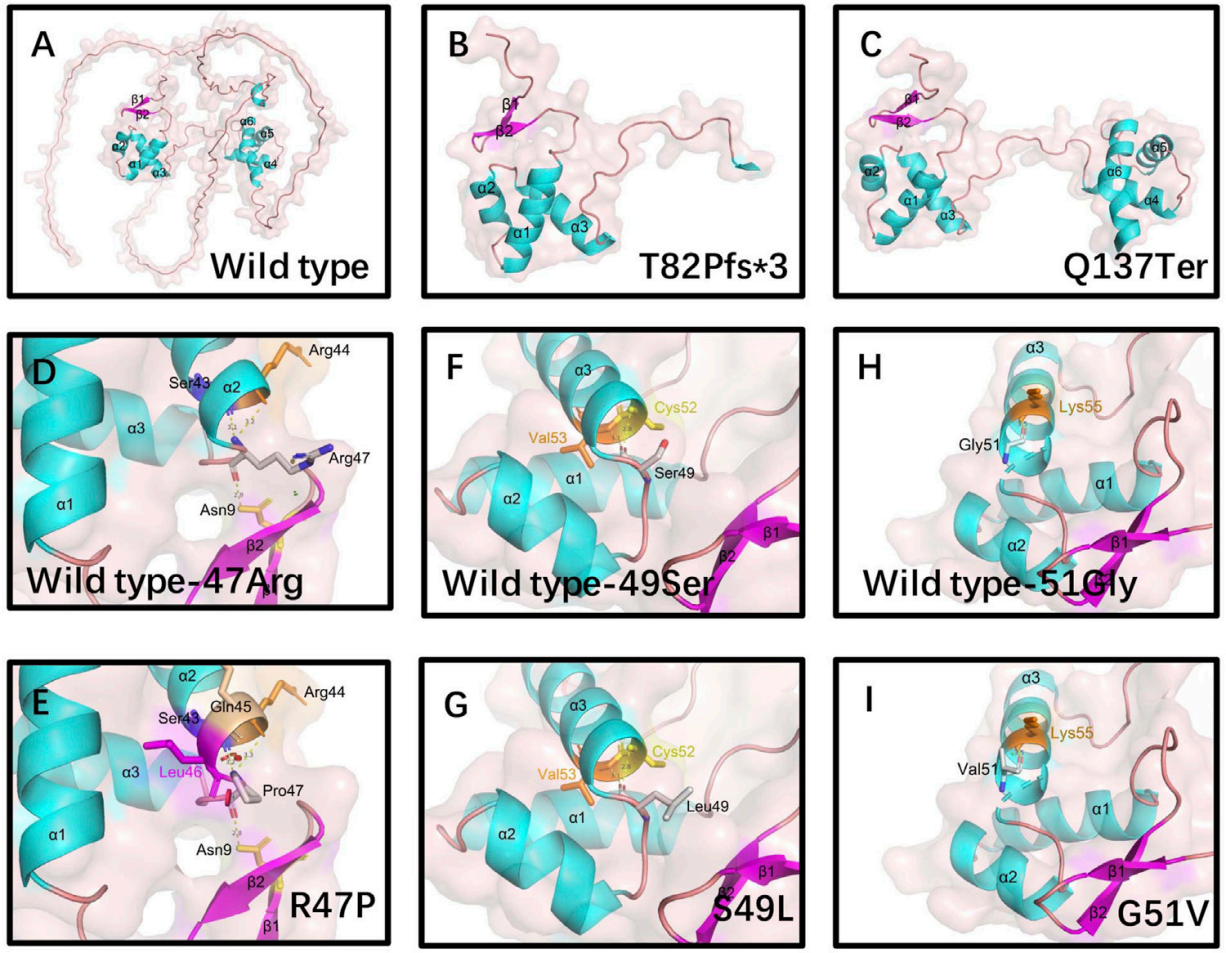
Structural analysis of wild-type and variants of PAX9 proteins. **(A)** wild-type PAX9 protein, **(B-C)** variant PAX9 p.T82fs*3 and p.Q137Ter protein. Hydrophobic Pro47 **(E)** introduces a Van der Waals clashes with Gln45 and Leu46(red disc) when compared with wild-type positively charged Arg47 **(D)**. Hydrophilic residue Ser49 **(F)** changed into a hydrophobic residue Leu49 **(G)**. Hydrophilic residue Gly51 **(H)** changed into hydrophobic Val51 **(I)**.

PAX9 contains a PD and an octapeptide motif. The wild-type PD is represented by an N-terminal subdomain (composed of β1, β2-sheets, and α1, α2, α3 helices) and a C-terminal subdomain (composed of α4, α5, α6 helices) connected by a linker (Figure 3A). 3D structural analysis was performed to assess the conformational changes of the PAX9 variants and their impact on protein function.

Compared to the wild type, the Thr82Profs*3 variant resulted in the absence of the N-terminal subdomain (Figure 3B). The Gln137Ter variant caused truncation of the PAX9 protein at the end of the PD (Figure 3C). The Arg47Pro variant may introduce a Van der Waals interaction between Gln^45^ and Leu^46^ (Figures 3D, E), and analysis using I-Mutant 2.0 (Capriotti et al., 2005) showed a large decrease in the stability of this variant (ΔΔG (folding free energy) = −1.20 kcal/mol). The ΔΔG value is calculated from the unfolding Gibbs free energy value of the mutated protein minus the unfolding Gibbs free energy value of the wild type; a ΔΔG < −0.5 demonstrates a large decrease in stability. The Ser49Leu variant changes an uncharged polar residue to a non-polar residue (Figures 3F, G), and I-Mutant analysis showed a large decrease in the stability of this variant (ΔΔG value = −0.57 kcal/mol). Although the Gly51Val variant does not change physicochemical properties, the volume of the side chain is augmented, and I-Mutant analysis showed a large decrease in the stability of this variant (ΔΔG value = −0.90 kcal/mol) (Figures 3H,I).

### 3.3 Novel PAX9 variants affected the nuclear localization and decreased protein expression

**FIGURE 4:**
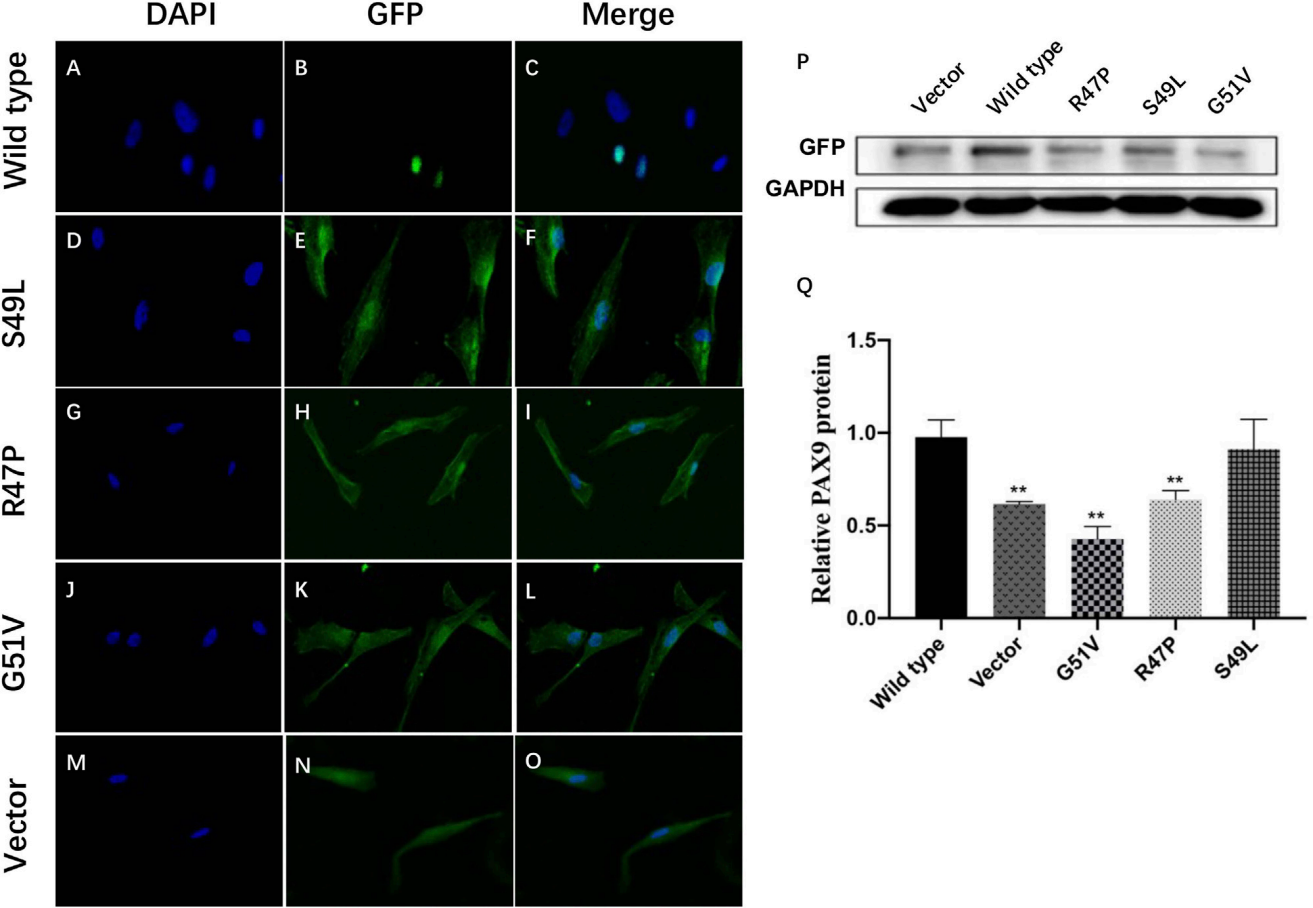
Functional studies of wild-type and variant PAX9 proteins. **(A–O)** subcellular localization of wild-type and variant PAX9 proteins evaluated by immunofluorescence assay. **(P, Q)** Whole-cell expression of wild-type and variant PAX9 proteins detected by Western Blotting ** *p* < 0.05.

PAX9 is normally located in the nucleus. Immunofluorescence showed that the Ser49Leu, Arg47Pro, and Gly51Val variants were expressed in both the nucleus and cytoplasm of transiently transfected hDPSCs; whereas wild-type PAX9 was only located in the nucleus (Figures 4A–O).

Western blot analysis showed that wild-type and three variants of PAX9 fusion proteins could be expressed in hDPSCs *in vitro*. The expression levels of variant proteins Arg47Pro and Gly51Val were significantly decreased compared with wild-type proteins (*p* < 0.001; Figures 4P, Q).

### 3.4 Genotype-phenotype analysis of patients with *PAX9* variants

The genotypes of 193 patients with 74 *PAX9* variants are shown in Supplementary Table S1 (Stockton et al., 2000; Nieminen et al., 2001; Das et al., 2002; Frazier-Bowers et al., 2002; Boyadjiev et al., 2003; Das et al., 2003; Lammi et al., 2003; Mostowska et al., 2003; Jumlongras et al., 2004; Klein et al., 2005; Zhao et al., 2005; Kapadia et al., 2006; Mostowska et al., 2006; Hansen et al., 2007; Tallón-Walton et al., 2007; Zhao et al., 2007; Guala et al., 2008; Wang et al., 2009a; Wang et al., 2009b; Haldeman-Englert et al., 2010; Pawlowska et al., 2010; Bergendal et al., 2011; Mendoza-Fandino et al., 2011; Paixão-Côrtes et al., 2011; Suda et al., 2011; Wang et al., 2011; Liang et al., 2012; Wang et al., 2012; Zhu et al., 2012; Mostowska et al., 2013a; Arte et al., 2013; Mostowska et al., 2013b; Boeira and Echeverrigaray, 2013; Mitsui et al., 2014; Thimmegowda et al., 2015; Haddaji Mastouri et al., 2016; Liang et al., 2016; Shahid et al., 2016; Yu et al., 2016; Choi et al., 2017; Daw et al., 2017; Murakami et al., 2017; Wong et al., 2018; Sun et al., 2021). We found that *PAX9*-related NSO accounted for 78.2% of the 193 patients, non-syndromic hypodontia for 18.1%, and syndromic oligodontia account for 2.6%. The same variant could lead to diverse phenotypes. Agenesis could affect all types of permanent teeth, while in deciduous dentition, only primary molars were affected, and all patients with agenesis of primary teeth had truncated PAX9 variants, such as frameshift and non-sense variants.

**FIGURE 5:**
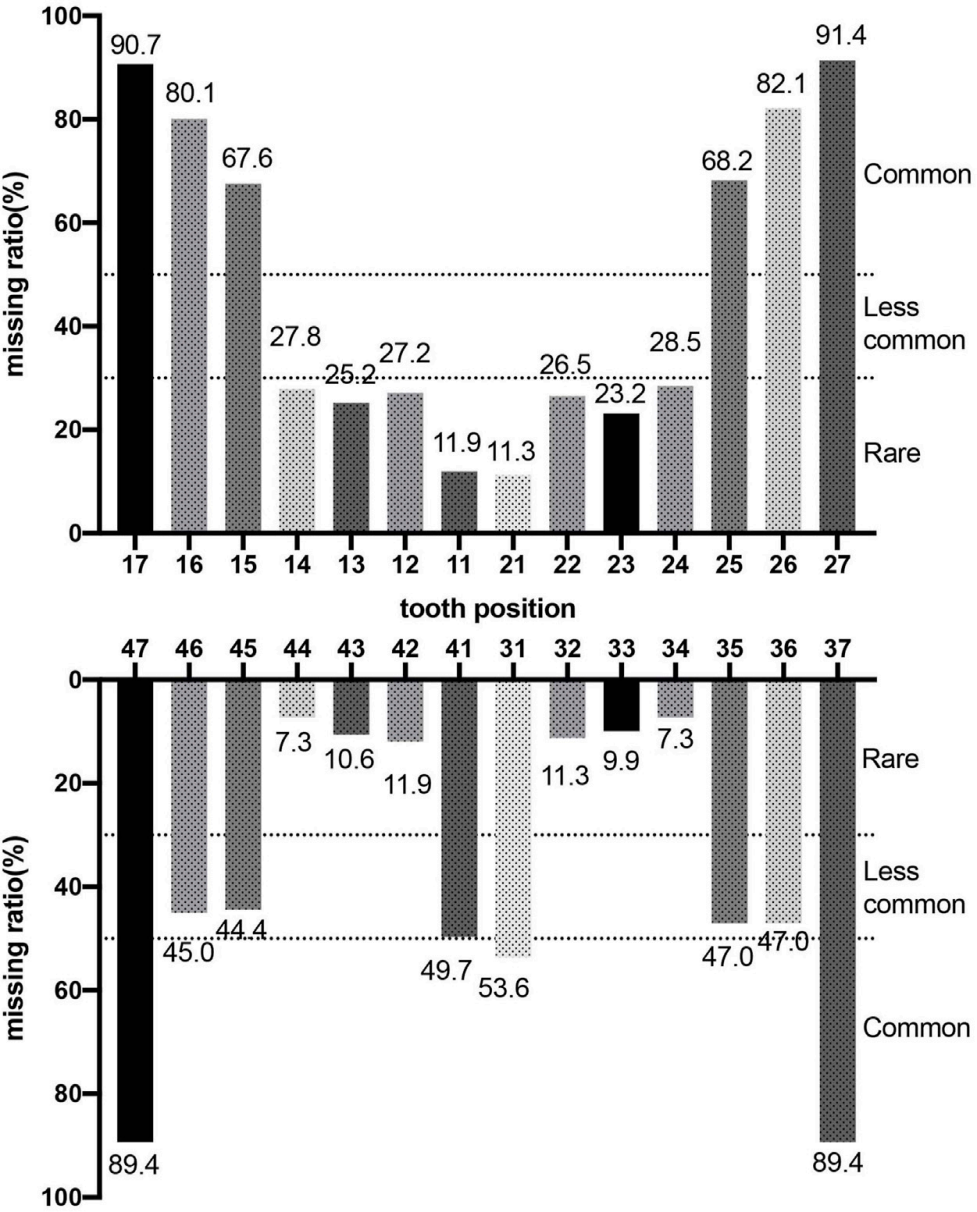
Percentage of missing teeth in patients with non-syndromic oligodontia (*n* = 151) caused by *PAX9* gene variants.

**FIGURE 6:**
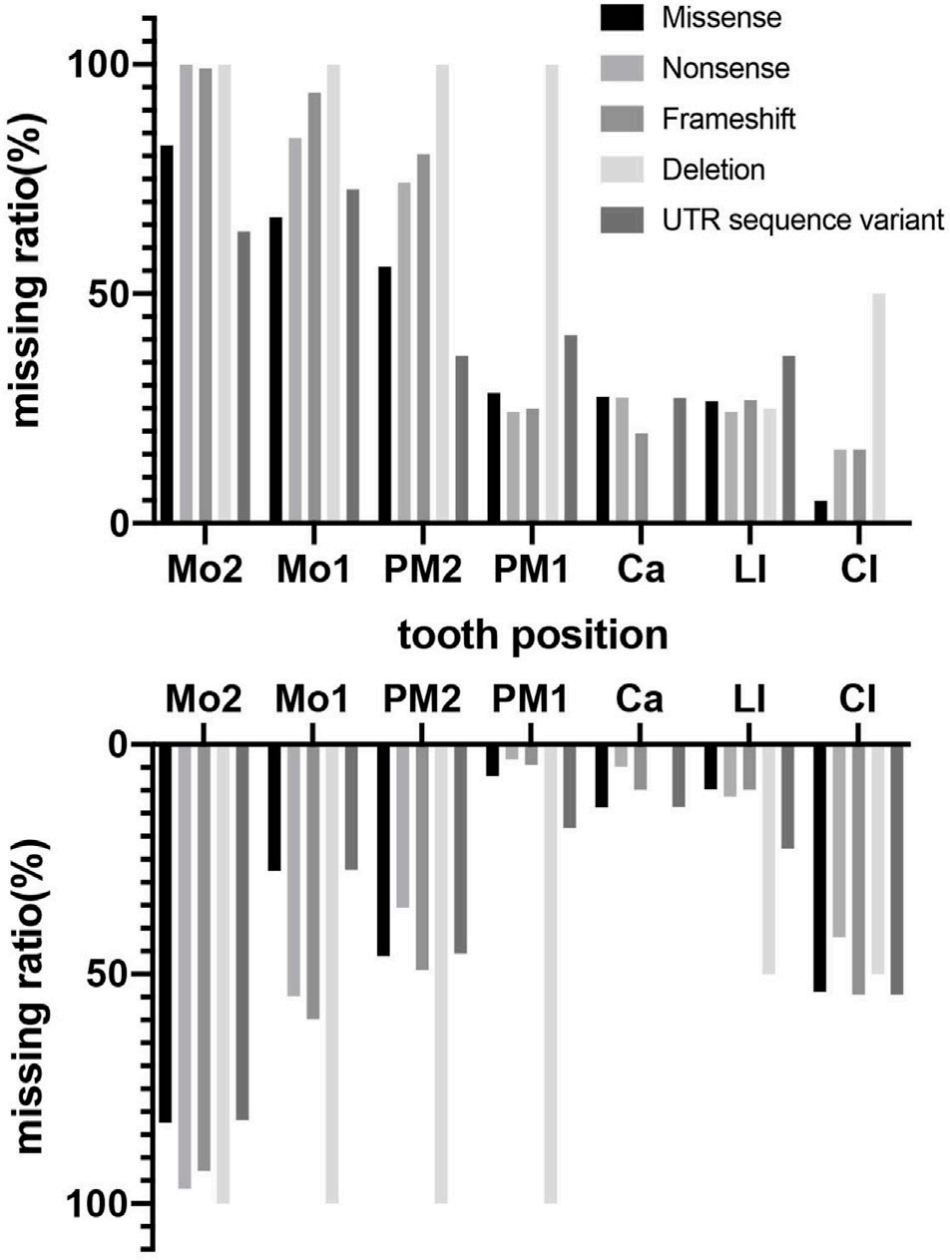
Percentage of missing teeth with different variant type in maxillary and mandibular arches in patients with non-syndromic oligodontia (*n* = 151) caused by *PAX9* gene variants.

**FIGURE 7:**
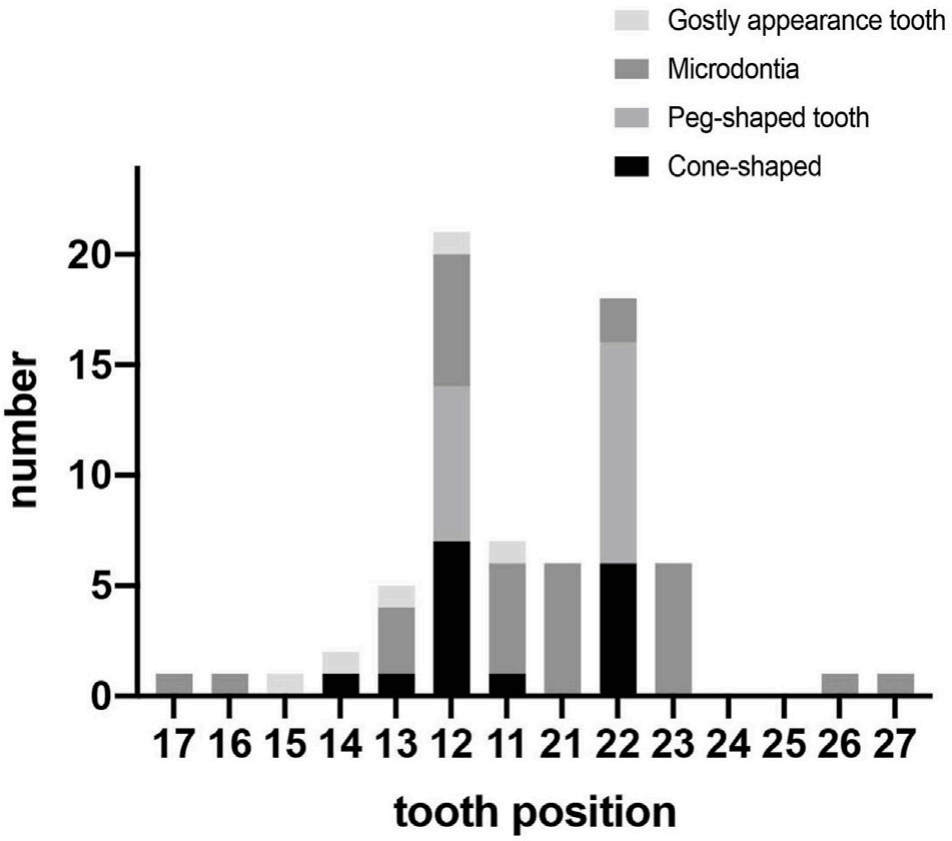
Number of teeth with abnormal morphology in permanent dentition in patients with non-syndromic oligodontia (*n* = 151) caused by *PAX9* gene variants.

Phenotypes of *PAX9*-related NSO were analyzed in detail. The ratio of missing teeth per tooth position in both upper and lower permanent dentitions in patients of NSO (*n* = 151) are shown in Figure 5. The incidence of commonly congenital agenesis tooth position was as follows: maxillary second molars > mandibular second molars > maxillary first molars > maxillary second premolars > mandibular central incisors. The ratio of missing teeth per type by missense, frameshift, non-sense, deletion, and UTR variants was shown in Figure 6. Two-way ANOVA showed no significant differences in missing tooth ratio among missense, non-sense, and frameshift variants, but there was a significant difference between deletion and the other three types of variants (F (4, 24) = 2.977, *p* = .0396), and between different permanent tooth positions (F (6, 24) = 15.66, *p* < .0001). In addition, microdontia, peg-shaped, and cone-shaped teeth were distributed mainly in the maxilla, especially in the lateral and central incisors (Figure 7).

## 4 Discussion

Tooth development is a complex process that encompasses sequential and reciprocal interactions between ectoderm-derived oral epithelium and neural crest-derived mesenchyme; and involves several genes, growth factors, transcription factors, and extracellular matrix molecules (Balic and Thesleff, 2015). *PAX9* is one of the most widely studied genes in odontogenesis. *PAX9* belongs to the paired box gene family and encodes a transcription factor that recognizes target DNA via a DNA-binding PD (Peters et al., 1998b). The PD (1st to 136th amino acid) is highly conserved with a bipartite structure and N-terminal and C-terminal subdomains. In 2017, Bonczek et al. reviewed all reported PAX9 variants associated with tooth agenesis, and found 26 out of 52 variants clustered in the PD (Bonczek et al., 2017), which implies that PD is a variant hotspot. Despite these advances, the exact pathogenic mechanisms underlying *PAX9*-related NSO have not yet been elucidated.

Our study identified three novel variants of *PAX9*: c.152G>T (p.Gly51Val), c.239delC (p.Thr82Profs*3), and c.409C>T (q.Gln137Ter), as well as two known variants: c.140G>C (p.Arg47Pro) and c.146C>T (p.Ser49Leu) in five unrelated Chinese Han families with NSO. The probands showed similar phenotypes with tooth agenesis predominantly in the molar region. The three missense variants and c.239delC are located within the PD. C.409C>T is a non-sense variant resulting in premature termination of the PAX9 protein synthesis at the 137th amino acid immediately after the PD. Consistent with previous studies, this study also confirmed that the PD region is the germline variant hotspot (Wong et al., 2018) and the main functional region of PAX9 in mediating odontogenesis (Sun et al., 2021).

*In silico* analysis predicted that the five variants were pathogenic, and homology analysis showed that the three missense mutated amino acids were highly conserved among eight vertebrates and Pax orthologs. Conformational analysis suggested impaired DNA binding ability of the truncated protein Thr82Profs*3, which lacks the C-subdomain, because sequence-specific recognition of DNA targets by PD is achieved through the coordinated use of both the N- and C-subdomains (Mensah et al., 2004). The c.409C>T variant introduces a premature stop codon in exon 3 and encodes a truncated protein containing the entire PD, which may reduce the transcriptional activity of PAX9 owing to the loss of a C-terminal transactivation domain (Zhu et al., 2012). We identified significant structural changes in PD around helix-α3 and the ΔΔG changes of mutant sequences showed a dramatic reduction in protein stability of the three missense substitutions Arg47Pro, Ser49Leu, and Gly51Val. Therefore, we predicted that these structural alterations could affect the DNA binding activities of the mutant protein, thereby affecting the expression levels of downstream signaling molecules in tooth development.

We performed functional analyses of the PAX9 variants to explain the oligodontia phenotype. Western blotting showed that all analyzed variants, Arg47Pro, Ser49Leu, and Gly51Val, were stably expressed in hDPSCs *in vitro*. Our immunofluorescence assay showed that all PAX9 variants were mainly expressed in the cytoplasm. Ser49Leu showed a small amount of expression in the nucleus, while wild-type PAX9 was translocated to the nucleus. These results were consistent with our *in silico* analysis results. The immunofluorescence results of Sun et al. also showed decreased nuclear localization with the frameshift PAX9 variant T80Lfs*6 transiently transfected 293T cells (Sun et al., 2021). We, therefore, speculated that the reduction of PAX9 in the nucleus caused by these variants may lead to lower binding and regulation of downstream targets (Sun et al., 2021).

To date, more than 70 *PAX9* variants have been reported with varying phenotypes of tooth agenesis, from microdontia and hypodontia to the most common oligodontia. Our genotype-phenotype analysis suggests that *PAX9* variants mainly cause autosomal-dominant NSO (Sun et al., 2021), occasionally non-syndromic hypodontia, and seldom correlate with syndromic tooth agenesis (Mostowska et al., 2013b). We also suggested that *PAX9* exhibits very high genetic heterogeneity since the same variant can lead to diverse tooth agenesis phenotypes (Bonczek et al., 2017). Considering the pattern of missing teeth, we found *PAX9* variants lead to the absence of molars, predominantly the second molars (above 89%). The first premolar in the mandibular is least affected in addition to the central incisor in the maxillary, which is largely consistent with the results of Fournier et al. (Fournier et al., 2018), which included 31 *PAX9* variants and 132 patients. The larger group (total 74 *PAX9* variants, 193 patients) we analyzed may have led to the slight difference in the absence rate of mandibular teeth. The maxillary incisors are the most susceptible to abnormal tooth morphology, suggesting the importance of normal PAX9 expression in regulating the tooth shape of the maxillary incisors. Our results reveal that phenotype severity is associated with the type of variant since deletion of PAX9 lead to higher numbers of missing permanent teeth and are often accompanied by primary molar agenesis (Jumlongras et al., 2004). Missense variants lead to oligodontia of permanent teeth with unaffected primary dentition, supporting the view that the *PAX9* variant phenotype is dosage-dependent (Fournier et al., 2018; Wong et al., 2018). This was confirmed by an *in vitro* experiment that showed that the level of Pax9 in transgenic mice was inversely proportional to the number of missing teeth (Kist et al., 2005). A detailed summary relating these clinical phenotypes may facilitate future genetic counseling.

In conclusion, we identified a novel frameshift variant c.239delC (p.Thr82Profs*3), a new non-sense variant c.409C>T (p.Gln137Ter), and a new missense variant c.152G>T (p.Gly51Val) in the PAX9 gene among Chinese Han families with sporadic NSO. In addition, we summarized 64 PAX9 variants genotype-phenotype correlations in 151 NSO patients and found that the most dominant feature was the high incidence of congenital missing upper second and first molars, upper second premolars, and lower second molars. Additionally, deletion variants caused a significantly higher congenital teeth missing ratio than missense, non-sense, and frameshift variants did. Furthermore, we performed preliminary bioinformatic and functional studies and confirmed that PAX9 structural changes contributed to NSO in patients with *PAX9* variants. This allowed us to expand the spectrum of *PAX9* mutations and provide a genetic basis for the pathogenesis of congenital tooth agenesis. This research could help in preconception genetic counseling, prenatal screening, and fetal diagnosis, which could contribute to disease status prediction in NSO families.

## Data Availability

The datasets presented in this study can be found in online repositories. The names of the repository/repositories and accession number(s) can be found below: https://www.ncbi.nlm.nih.gov/, Submission ID:SUB9478476; Accession:SCV001571248 https://www.ncbi.nlm.nih.gov/, Submission ID: SUB9482070; Accession: SCV001571253 https://www.ncbi.nlm.nih.gov/, Submission ID: SUB9482163; Accession: SCV001571255 https://www.ncbi.nlm.nih.gov/, Submission ID: SUB9482223; Accession: SCV001571257 https://www.ncbi.nlm.nih.gov/, Submission ID: SUB9482232; Accession: SCV001571258.

## Footnotes

1
https://bite-it.helsinki.fi.

2
https://www.ncbi.nlm.nih.gov/tools/cobalt/re_cobalt.cgi.

3
http://gpcr2.biocomp.unibo.it/cgi/predictors/I-Mutant3.0/I-Mutant3.0.cgi.
